# Pericardial Involvement in ST-Segment Elevation Myocardial Infarction as Detected by Cardiac MRI

**DOI:** 10.3389/fcvm.2022.752626

**Published:** 2022-02-24

**Authors:** Eias Massalha, Yafim Brodov, Daniel Oren, Alex Fardman, Sharon Shalom Natanzon, Israel Mazin, Roy Beinart, Ronen Goldkorn, Eli Konen, Elio Di Segni, Amit Segev, Roy Beigel, Shlomi Matetzky, Orly Goitein

**Affiliations:** ^1^The Olga and Lev Leviev Heart Center, Sheba Medical Center, Affiliated With the Sackler School of Medicine, Tel Aviv University, Ramat Gan, Israel; ^2^Diagnostic Imaging, Sheba Medical Center, Affiliated With the Sackler School of Medicine, Tel Aviv University, Ramat Gan, Israel; ^3^Division of Cardiology, Department of Medicine, College of Physicians and Surgeons, Columbia University Vagelos, New York, NY, United States

**Keywords:** ST-segment elevation myocardial infarction, post myocardial infarction pericarditis, late Gadolinium enhancement, late pericardial enhancement, cardiac MRI, prognosis

## Abstract

**Background:**

Post myocardial infarction pericarditis is considered relatively rare in the current reperfusion era. The true incidence of pericardial involvement may be underestimated since the diagnosis is usually based on clinical and echocardiographic parameters.

**Objectives:**

This study aims to document the incidence, extent, and prognostic implication of pericardial involvement in ST-segment elevation myocardial infarction (PISTEMI) using cardiac MRI (CMR).

**Methods:**

One hundred and eighty-seven consecutive ST-segment elevation myocardial infarction patients underwent CMR on day 5 ± 1 following admission, including steady-state free precession (SSFP) and late Gadolinium enhancement (LGE) sequences. Late Gadolinium enhancement and microvascular obstruction (MVO) were quantified as a percentage of left ventricular (LV) mass. Late Gadolinium enhancement was graded for transmurality according to the 17 AHA left ventricle (LV) segment model (LGE score). Late pericardial enhancement (LPE), the CMR evidence of pericardial involvement, was defined as enhanced pericardium in the LGE series and was retrospectively recorded as present or absent according to the 17 AHA segments. Late pericardial enhancement was evaluated adjacent to the LV, the right ventricle, and both atria. Clinical, laboratory, angiographic, and echocardiographic data were collected. Clinical follow-up for major adverse cardiac events (MACE) was documented and correlated with CMR indices, including LGE, MVO, and LPE.

**Results:**

Late pericardial enhancement (LPE+) was documented in 77.5% of the study cohort. A strong association was found between LPE and the degree and extent of myocardial injury (LGE, MVO). Both LGE and MVO were significantly correlated with increased MACE on follow-up. On the contrary, LPE presence, either adjacent to the LV or the other cardiac chambers, was associated with a lower MACE rate in a median of 3 years of follow-up HR 0.39, 95% CI (0.21–0.7), *p* = 0.002, and HR 0.48, 95% CI (0.26–0.9), *p* = 0.02, respectively.

**Conclusions:**

Prognostic implication of pericardial involvement in ST-segment elevation myocardial infarction was documented by CMR in 77.5% of our STEMI cohort. Late pericardial enhancement presence correlated significantly with the extent and severity of the myocardial damage. Unexpectedly, it was associated with a considerably lower MACE rate in the follow-up period.

## Introduction

Post-myocardial infarction pericarditis occurs within several days following ST-elevation myocardial infarction (STEMI) (1–4). Its reported incidence has decreased dramatically from 20% to <5% since the early reperfusion era (1–6). Most of the reports addressing its incidence were based on the combination of clinical, electrocardiographic, and echocardiographic data (1–6).

The current practice of CMR performed early in the post-STEMI period suggested that pericardial involvement is more frequent than previously reported. This led us to conduct a dedicated, early post-STEMI CMR study to document the actual incidence and clinical significance of pericardial involvement. Cardiac MRI using the late Gadolinium enhancement (LGE) sequence is the modality of choice for assessing myocardial and pericardial pathologies (7, 8). The reported sensitivity and specificity of CMR for detecting pericardial involvement is 94–100% (9). Thus, CMR is superior to echocardiography in assessing and characterizing both myocardium and pericardium (9).

The current study documents the incidence, location, and extent of pericardial involvement using CMR in consecutive STEMI patients (PISTEMI). Clinical follow-up was conducted as well in order to evaluate the long-term clinical significance of PISTEMI.

## Methods

### Study Population

The study includes a single-center cohort of 187 consecutive patients who presented with a first STEMI. All study patients underwent primary percutaneous coronary intervention within 12 h of symptoms onset. Demographic characteristics, co-morbidities, pain-to-balloon time, ECG characteristics including MI location, and ΣST-segment elevation at presentation were documented. Laboratory results collected included serial troponin, creatine phosphokinase (CPK), and C-reactive protein (CRP) levels.

Echocardiographic and angiographic parameters were recorded as well. The Institutional Ethics Review Board approved the study.

### Cardiac MRI

All patients underwent cardiac MRI (CMR) on day 5 ± 1 following admission. Cardiac MRI was performed using a 1.5-T magnet (General Electric, Optima mr450w GEM versionDV26) (*N* = 101/187 patients) and a 3-T magnet (Philips Ingenia 3T version 5.4.1.2) (*N* = 86/187 patients) according to scanner availability. Conventional CMR sequences included: balanced steady-state free precession (B-SSFP) cine imaging for cardiac function evaluation and LGE imaging for tissue characterization. Late Gadolinium enhancement was performed 8–12 min following the intravenous administration of Gadolinium-based contrast agent (0.1 mmol/kg of Gadoterate meglumine, Guerbet, S.A. France). **A phase-sensitive inversion recovery technique was used. The in-plane resolution of the LGE images was 2 mm**. Late Gadolinium enhancement sequences were performed with and without fat suppression to distinguish between pericardial fat and pericardial enhancement.

Left and right ventricular ejection fractions (LVEF, RVEF), LGE%, and microvascular obstruction (MVO) % of LV mass were calculated in the short axis plane using a dedicated platform (Medis Medical Imaging version 7.6 Leiden, Netherlands). The LGE signal intensity threshold for scar quantification was set at five standard deviations (SD) above the reference ROI in the remote unaffected myocardium (10). This threshold was used for the quantification of both LGE% and MVO%. The segmental left ventricle (LV) myocardial analysis was performed according to the AHA 17-segment model (11). The extent of myocardial involvement (according to LGE presence) per segment was graded as follows: grade 0: no LGE, grade 1: 1–25%, grade 2: 26–50%, grade 3: 51–75%, and grade 4: 76–100% of the myocardial wall thickness. Late Gadolinium enhancement score was calculated by summing all segmental LGE grades. The presence or absence of MVO was documented using the AHA 17-segments model per myocardial segment. Microvascular obstruction score was calculated by the sum of all segments with MVO.

**Figure 1:**
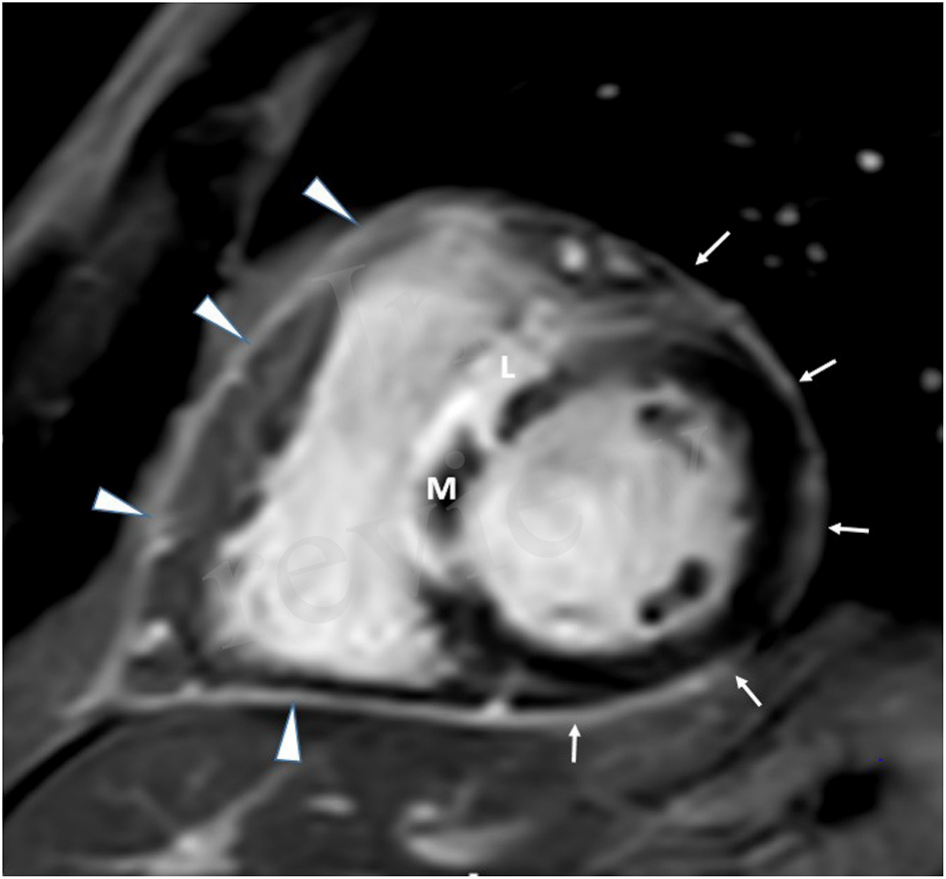
CMR in a patient with extensive anterior MI-short axis view.

**Figure 2:**
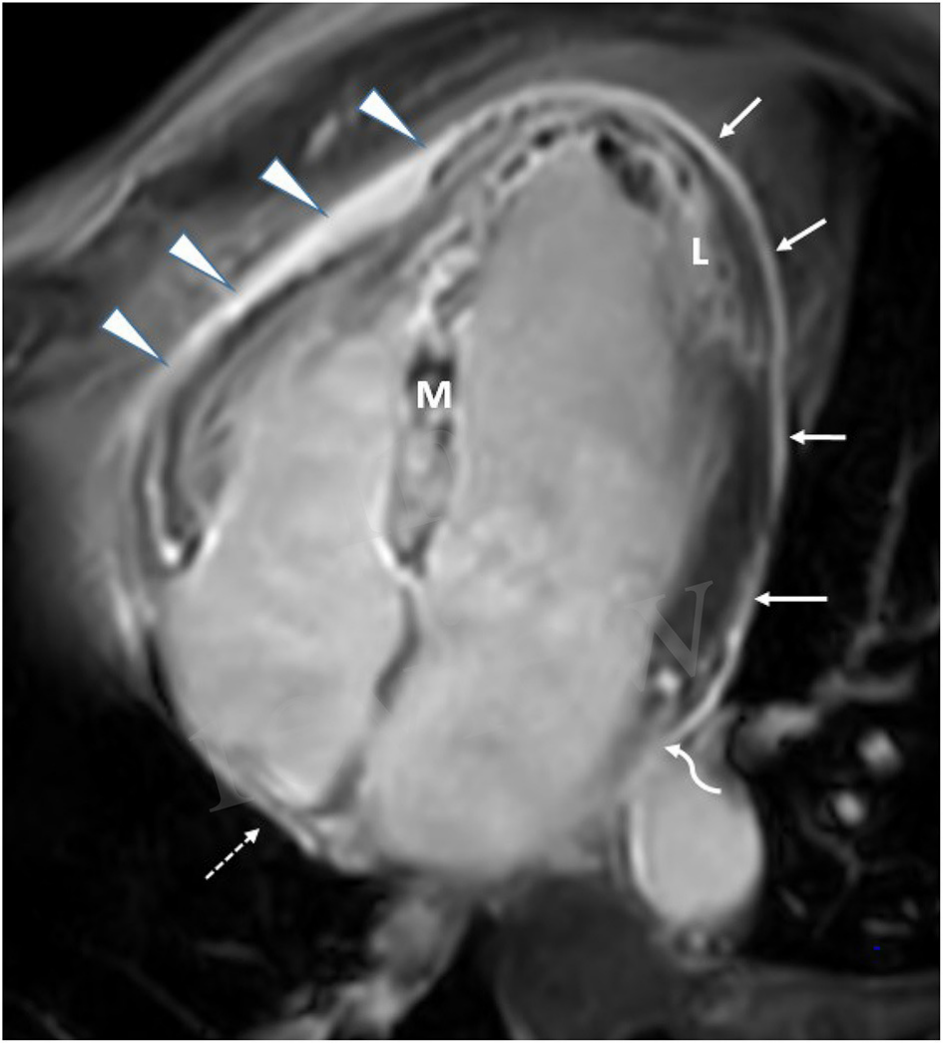
CMR in a patient with extensive anterior MI-four-chamber view. Late Gadolinium enhancement (L), demonstrating anteroseptal and inferolateral transmural MI with microvascular obstruction (M). Extensive late pericardial enhancement (LPE) is demonstrated involving the left ventricle (white arrows), right ventricle (arrowhead), right atrium (dotted arrow), and left atrium (curved arrow).

Late pericardial enhancement (LPE) was retrospectively evaluated and was defined as the presence or absence of enhanced pericardium on the LGE series (Figures 1, 2). **Late Gadolinium enhancement series were obtained with and without fat suppression and were used to differentiate LPE from fat in the pericardium**. Qualitative assessment of pericardial enhancement on the LGE images (LPE) was evaluated visually as present or absent in the left ventricular (LV), right ventricular (RV), right atrial (RA), and left atrial (LA) walls. For the LV, LPE was analyzed using the AHA 17 segment model, excluding the septal segments and apex segments 2, 3, 8, 9, 14, 17). The septal segments were excluded since anatomically, the pericardium does not cover the septum. The LPE adjacent to the RV, RA, or LA was considered a single segment for each chamber. Late pericardial enhancement score was calculated by summing all involved pericardial segments (LPE+) in the LV, RV RA, and LA. Late pericardial enhancement was evaluated in a control group of 34 consecutive patients referred for evaluation of non-acute pathologies, including [cardiomyopathy (*n* = 25), RV dysplasia (*n* = 1), viability (*n* = 5), and LV non-compaction (*n* = 1)]. Late pericardial enhancement in this cohort was documented in only two patients (2/34, 5.8%) and only four pericardial segments (4/479, 0.8%).

The correlation between LPE presence and different clinical, laboratory, and CMR parameters was examined. Late pericardial enhancement segmental location was correlated with segmental distribution and the extent of the myocardial damage (LGE and MVO scores).

Pericardial effusion on CMR was determined using SSFP in the four-chamber plane and was defined as pericardial width ≥4 mm (3).

The current study did not include T2 sequences in evaluating pericardial involvement, as LPE is better suited for evaluating the focal pericardial involvement in STEMI, as compared with T2 sequences. Post-processing for LGE, MVO, and LPE was performed by two experienced cardiovascular imagers (OG-16 years of experience and YB-9 years of experience).

### Post-MI Pericarditis: An Integrated Approach

In order to establish the clinical significance of the CMR findings demonstrating pericardial involvement (PISTEMI), we set out to integrate it together with other findings related to diagnosis of pericarditis (1, 3), including increased CRP levels and the presence of pericardial effusion (defined as effusion ≥4 mm on CMR). We assume that the combination of pericardial enhancement in addition to increased inflammatory indices or the presence of pericardial effusion indicates an active pericardial inflammatory process (pericarditis).

### Clinical Follow-Up

Clinical follow-up for major adverse cardiac events (MACE) was performed. Major adverse cardiac event included recurrent MI, acute stroke, acute coronary syndrome necessitating urgent hospitalization and\ or percutaneous coronary intervention, hospitalization for heart failure, and cardiovascular death in a median of 3 years of follow-up.

Kaplan-Meier survival analysis (Kaplan and Meier, 1958) was conducted to compare the incidence of MACE in patients with LPE vs. others. A similar analysis was conducted for patients with a left ventricle LPE (LV LPE) vs. others. A log-rank test was conducted to determine if there were differences in MACE incidence.

### Statistical Analysis

Statistical analysis was performed using the SPSS statistical software 25.0.0 (IBM, Armonk, NY, USA) and the R foundation statistical computing and graphics software (version 4.0.0). All statistical tests were two-sided, and a *p*-value of <0.05 was considered significant. Categorical variables are reported in frequencies and percentages. The significance of categorical variables between groups was assessed using the chi-square test or Fisher's exact test.

We tested all variables for normal distribution by the Kolmogorov-Smirnov test and visualized the QQ-plot, plotting the distribution and variance of the residuals. Normally distributed continuous variables were reported as mean and SD values, and differences between groups were assessed using the student's *t*-test. Continuous variables not normally distributed were reported as median and interquartile range (IQR, 25th−75th percentiles) values, and significance was assessed using the Mann-Whitney-Wilcoxon or Kruskal-Wallis tests. Interobserver variability for the presence or absence of LGE, MVO, and LPE was assessed using blinded data from the study cohort. Inter-observer variability demonstrated a Cohen's κ weighted score of 0.76 [*P* < 0.001. 95% CI (0.68, 0.84)], indicating a good inter-observer agreement.

The study cohort was divided into two groups according to the presence or absence of LPE on CMR. A multivariable binary logistic regression model was constructed for the prediction of pericardial involvement. The model consisted of variables that were statistically significant in univariate tests and of clinically relevant indices. Notably, variables with missing values for more than 15% of the cohort (CRP) were excluded from the multivariate analysis. A K-M survival curve and Cox proportional hazard regression analysis examined the CMR indices and MACE association.

## Results

**Figure 3:**
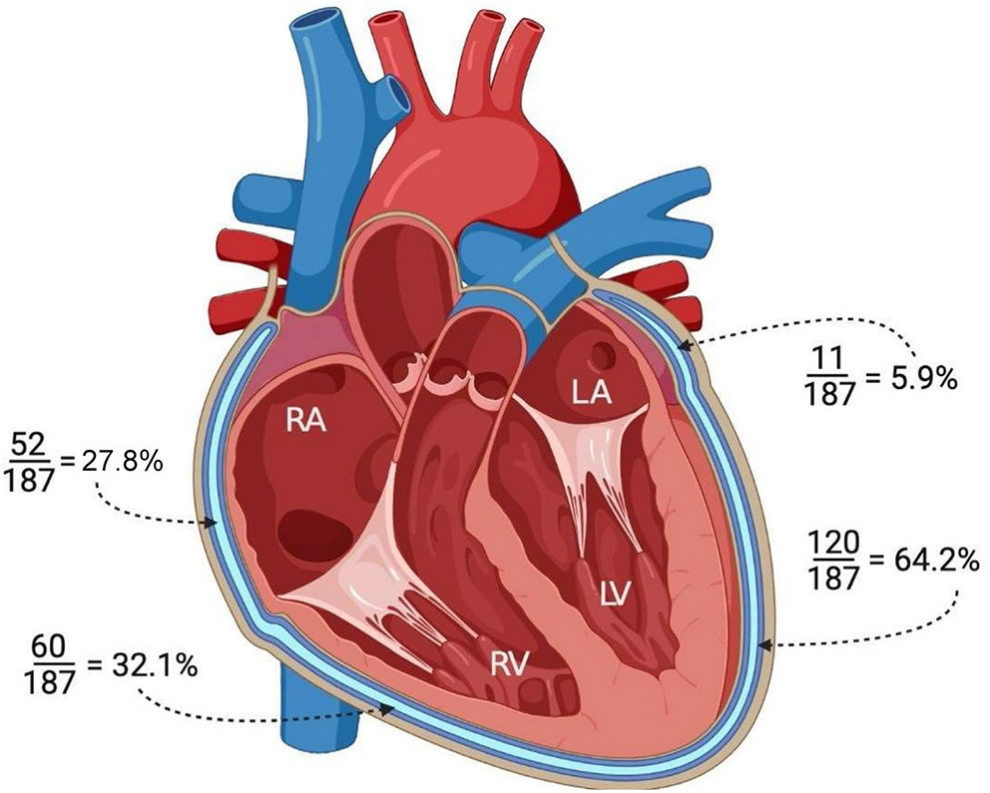
Illustration of the heart in the four-chamber view demonstrating the pericardial involvement (LPE+) adjacent to each cardiac chamber. LV, left ventricle; RV, right ventricle; RA, right atrium; LA, left atrium.

Involvement of the pericardium (LPE+) overlying the ventricles and\or the atria was demonstrated in 145/187 patients (77.5 %). Among these 145 patients, spatial pericardial involvement was demonstrated as follows: LPE overlying the LV (120/187, 64.2%), RV (60/187, 32.1%), RA (52/187, 27.8%) and LA (11/187, 5.9%) (Figure 3).

The study cohort was divided accordingly into LPE+ and LPE– subgroups. Baseline characteristics, laboratory and echocardiographic findings are presented in Table 1. No significant differences in demographics, baseline characteristics, co-morbidities, MI location, or ECG features were found between the two groups (Table 1, Supplementary Table 1).

**Table 1:** Baseline characteristics of patients with or without pericardial involvement on CMR.

	**All patients**	**LPE+**	**LPE–**	***P*-value**
	***N* = 187**	**145 (77.5%)**	**42 (22.5%)**	
Age, years, mean ± sd	57.2 ± 10.5	57.2 ± 10.4	57.2 ± 11.1	0.96
Male gender, *N* (%)	171 (91.4%)	135 (93.1%)	36 (85.7%)	0.23
Active smoker, *N* (%)	79 (42.2%)	63 (43.4%)	16 (38.1%)	0.66
Hypertension, *N* (%)	58 (31.0%)	41 (28.3%)	17 (40.5%)	0.19
Diabetes mellitus, *N* (%)	27 (14.4%)	19 (13.1%)	8 (19.0%)	0.47
Dyslipidemia, *N* (%)	73 (39%)	59 (40.7%)	14 (33.3%)	0.49
Family history of IHD, *N* (%)	60 (32.1%)	50 (34.5%)	10 (23.8%)	0.26
Pain to balloon (h, median [IQR])	2.5 [2–5]	2.5 [2–5]	3 [2–6]	0.50
Lateral MI, *N* (%)	61 (32.6%)	50 (34.4%)	11 (26.1%)	0.41
WBC count^*^, K/μl (median [IQR])	11.2 [8.9–14.0]	11.3 [9.2–14]	11.1 [7.7–13.8]	0.24
WBC count^*^, K/μl (mean ± sd)	11.7 ± 3.9	11.9 ± 4.8	11.0 ± 3.3	0.17
Maximal CRP^**^, mg/L, (median [IQR])	16.7 [5.4–35.8]	17.2[5.6–40.7]	7.3 [4.4–28.8]	0.12
Maximal CRP^**^, mg/L (mean ± sd)	34.6 ± 49.9	37.8 ± 53.4	19.7 ± 24.1	0.01
Maximal CPK (U/L, median [IQR])	1,534 [776–3,040]	1,700 [900–3,133]	1,067 [482–2,709]	0.03
Maximal TROPONIN (μl/L, median [IQR])	53 [17.3–80.0]	54 [19.7–80.0]	32 [8.4–89.0]	0.49
LVEF % on first Echocardiography post-PPCI, mean ± sd	45.45 ± 9.6	44.6 ± 9.2	48.1 ± 10.3	0.03

*CRP, C-reactive protein; PPCI, primary percutaneous coronary intervention; CPK, creatine phosphokinase*.

* *White blood count at admission*.

** *C-reactive protein values were available in 130/187 patients*.

LPE+ patients had higher peak CPK levels (1,700 [IQR: 900–3,133] vs. 1,067 [IQR: 482–2,709], *p* = 0.03) and a lower LVEF on the first echocardiogram performed following primary PCI (44.6% ± 9.6 vs. 48.1% ± 10.3, *p* = 0.03).

Clinical signs of pericarditis were documented and included: pericardial chest pain in 5/187 patients (2.6%), typical ECG changes in 4/187 patients (2.1%), and pericardial effusion at trans-thoracic echocardiography in 6/187 patients (3.2%). Thus, a total of 13/187 patients demonstrated at least two clinical signs consistent with the diagnosis of pericarditis. Out of these patients, two patients only were treated with Colchicine (Table 2).

**Table 2:** CMR characteristics of patients with or without pericardial involvement on CMR.

	**All patients**	**LPE+**	**LPE–**	***P*-value**
	***N* = 187**	**145 (77.5%)**	**42 (22.5%)**	
**Classical pericarditis criteria**				
Pericardial clinical pain, *N* (%)	5 (2.7%)	4 (2.8%)	1 (2.4%)	>0.99
Typical ECG changes, *N* (%)	4 (2.1%)	4 (2.8%)	0 (0%)	0.62
Pericardial effusion (echocardiography), *N* (%)	6 (3.2%)	5 (3.4%)	1 (2.4%)	>0.99
**Colchicine treatment**, ***N*** **(%)**	2 (1.1%)	2 (1.4%)	0 (0%)	>0.99
**CMR parameters**				
LVEF CMR, mean ± sd	56 ± 10.9	55 ± 11.1	59.1 ± 9.8	0.03
RVEF CMR, mean ± sd	53 ± 9.6	52.6 ± 10.1	54.3 ± 7.7	0.32
LGE% of LV mass median [IQR]	24 [15–35]	27 [16–35]	20 [10–32]	0.08
LGE score median [IQR]	12 [5.5–20]	14 [8–20]	8 [0–13.5]	<0.01
MVO% of LV mass median [IQR]	0.6 [0–3.2]	1 [0–3.4]	0 [0–2.0]	0.01
MVO score median [IQR]	2 [0–4]	2 [0–4]	0 [0–3]	<0.01
Pericardial effusion (CMR), *N* (%)	90 (48.1%)	69 (47.6%)	21 (50.0%)	0.68

*CMR, cardiac MRI; LVEF, left ventricular ejection fraction; RVEF, right ventricular ejection fraction; LV, left ventricle; LGE, late Gadolinium enhancement; MVO, microvascular obstruction*.

**Figure 4:**
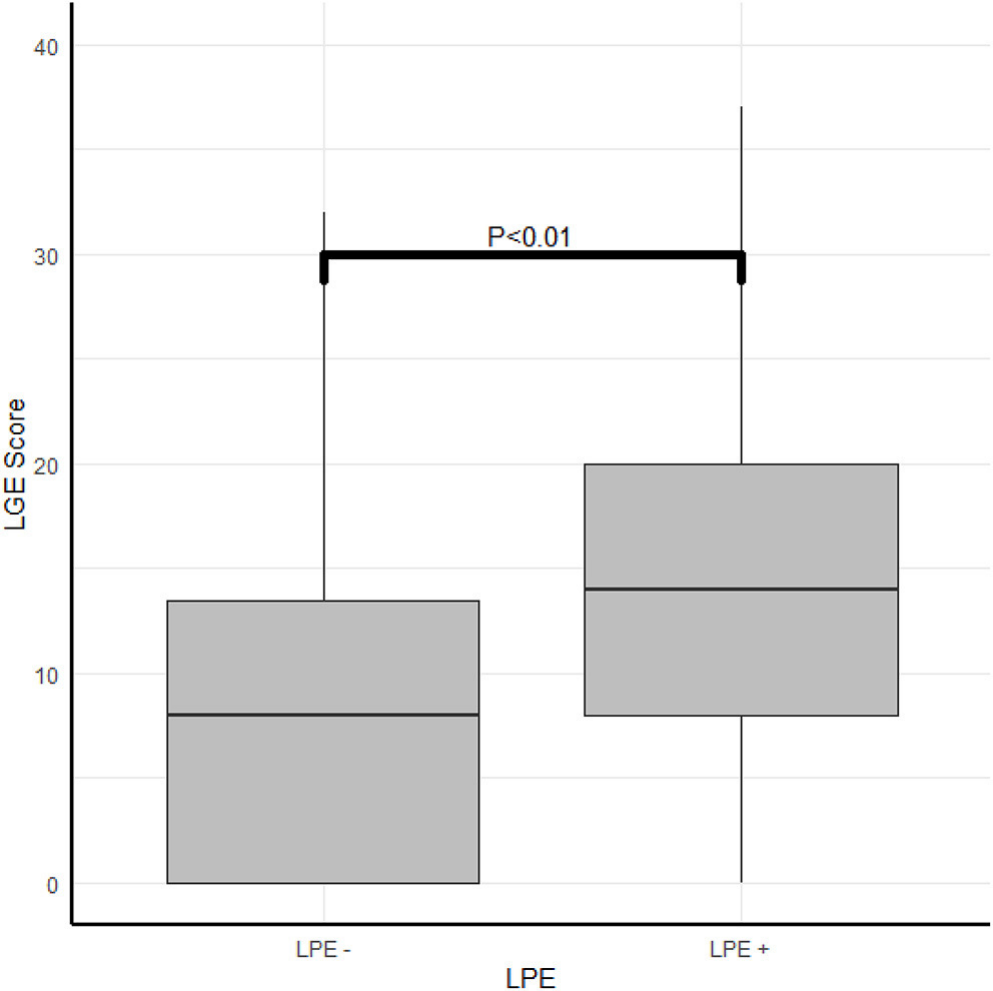
The correlation between myocardial damage and pericardial enhancement. The correlation between LPE and LGE score.

**Figure 5:**
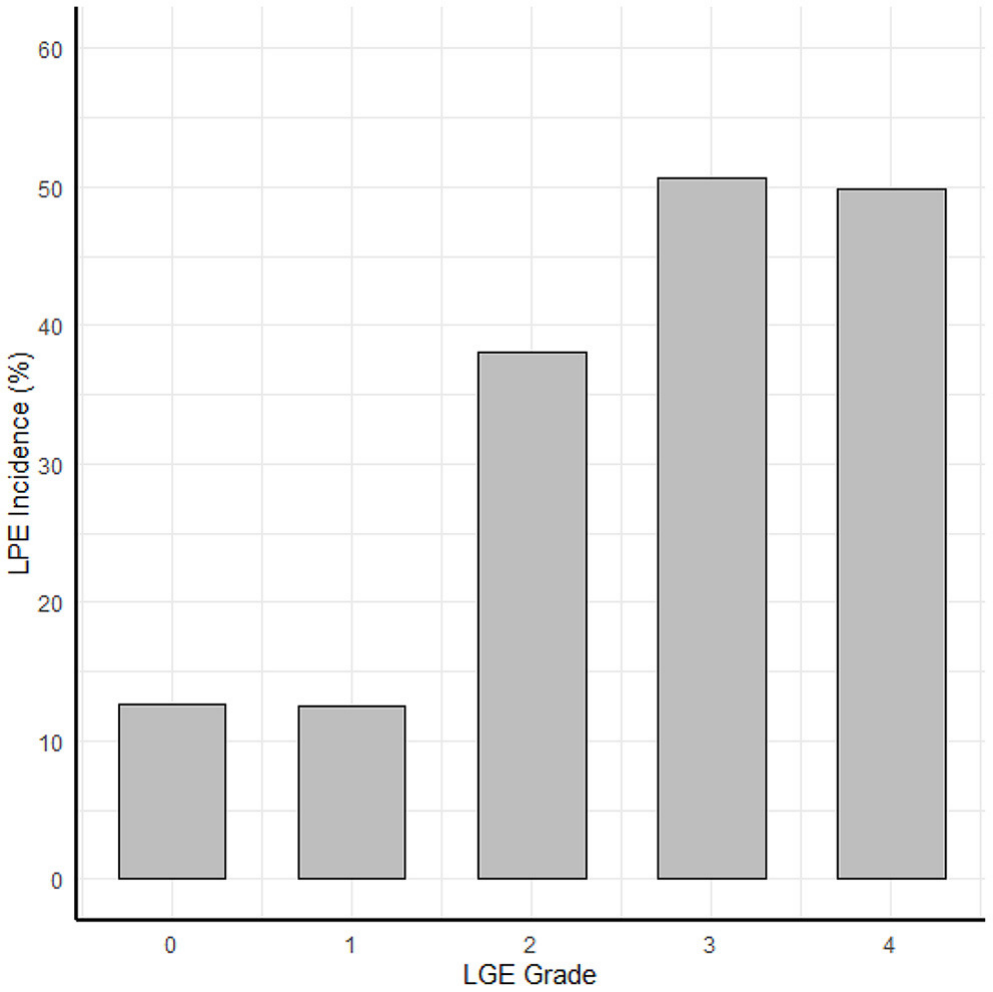
The correlation between myocardial damage and pericardial enhancement. The correlation between the LGE transmurallity (LGE grade in each segment) and LPE incidence (probability of LPE in that segment).

A robust association was found between the LGE score, expressing the degree and extent of the myocardial damage and the presence of LPE (*P* < 0.01) (Table 2; Figures 4, 5). Furthermore, a strong association was found between LPE overlying the left ventricle (LV LPE) and the severity of the myocardial damage expressed as LGE and MVO scores (*p* = 0.031 and *p* = 0.002, respectively).

No statistically significant correlation was found between the location of the myocardial damage and LPE presence. The prevalence of pericardial effusion detected by CMR was similar in the presence and absence of LPE (47.6 vs. 51.2%, *p* = 0.81) (Table 2). Moreover, the presence of pericardial effusion was not correlated with the LPE score (3 [IQR: 1–5] vs. 3 [IQR: 2–6], *p* = 0.55).

A binomial multivariable logistic regression model was performed to ascertain the effects of age, gender, LGE score, and MVO score on the likelihood of LPE+. Late Gadolinium enhancement score was found to be a robust predictor for pericardial involvement (*p* = 0.002). The logistic regression model was statistically significant [$\chi_{5}^{2}$ = 15.8, *p* = 0.007], and the area under the ROC curve was 0.74 (95% CI, 0.64–0.81) (Table 3). No significant difference in complications during hospitalization or length of stay was demonstrated between the LPE+ and LPE– groups (Table 1).

**Table 3:** Binomial multivariate logistic regression model for the different predictors of pericardial involvement (LPE+).

**Variables**	**Odds ratio**	**95% Confidence interval**	***P*-value**
Age, years	1.01	0.97–1.04	0.90
Male gender	1.93	0.59–6.23	0.27
LGE score	1.08	1.03–1.14	0.002
MVO% of LV mass	0.96	0.87–1.05	0.34

*LPE, late pericardial enhancement; LGE, late Gadolinium enhancement; MVO, microvascular obstruction*.

Post-discharge clinical follow-up was available in 85% of patients (159/187) for a median period of 3 years. Overall, 46/159 patients (28.9%) sustained at least one MACE, including recurrent MI (9/159, 5.6%), acute stroke (3/159, 1.8%), acute coronary syndrome necessitating urgent hospitalization and\or percutaneous coronary intervention (15/159, 9.4%), hospitalization for heart failure (18/159, 11.3%), and cardiovascular death (1/159, 0.6%). The presence and extent of LGE, MVO, and LPE in the follow-up patients were not different from those lost to follow-up. A detailed comparison of MACE between subgroups of the cohort can be found in the Supplementary Material (Supplementary Table 2).

Patients with a higher LGE score and MVO % of LV mass were more likely to sustain MACE during the follow-up period (43 vs. 22%, *p* = 0.006 and 40 vs. 23%, *p* = 0.024, respectively). Patients with LPE+ involving more than one pericardial segment demonstrated a lower MACE incidence than patients without pericardial enhancement (LPE–) (23 vs. 38%, *p* = 0.042). Pericardial involvement adjacent to the LV (LV LPE) was also associated with a significantly lower MACE rate (22 vs. 41%, *p* = 0.009). A univariate log-rank survival analysis revealed a statistically significant difference in survival distributions between LV LPE (+) and LV LPE (–) patients (*p* = 0.04). However, significance was not maintained when comparing whole myocardium LPE (*p* = 0.12).

**Figure 6:**
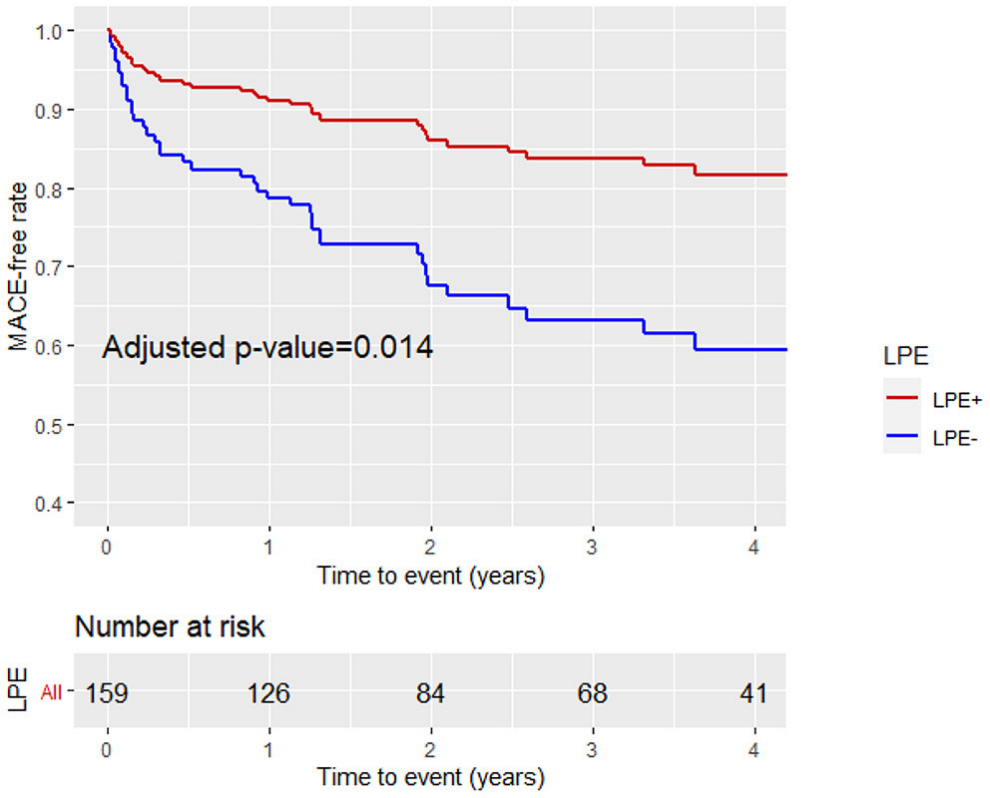
Kaplan-Meier curves analysis with the MACE-free rate on the y-axis (events) vs. time to event (years) on the x-axis stratified by pericardial involvement adjacent to any pericardial chamber (LV, RV, RA, LA) (LPE+, LPE–) after adjustment for age, gender, LGE, and MVO%. LPE, late pericardial enhancement; LV, left ventricle; RV, right ventricle; RA, right atrium; LA, left atrium.

**Figure 7:**
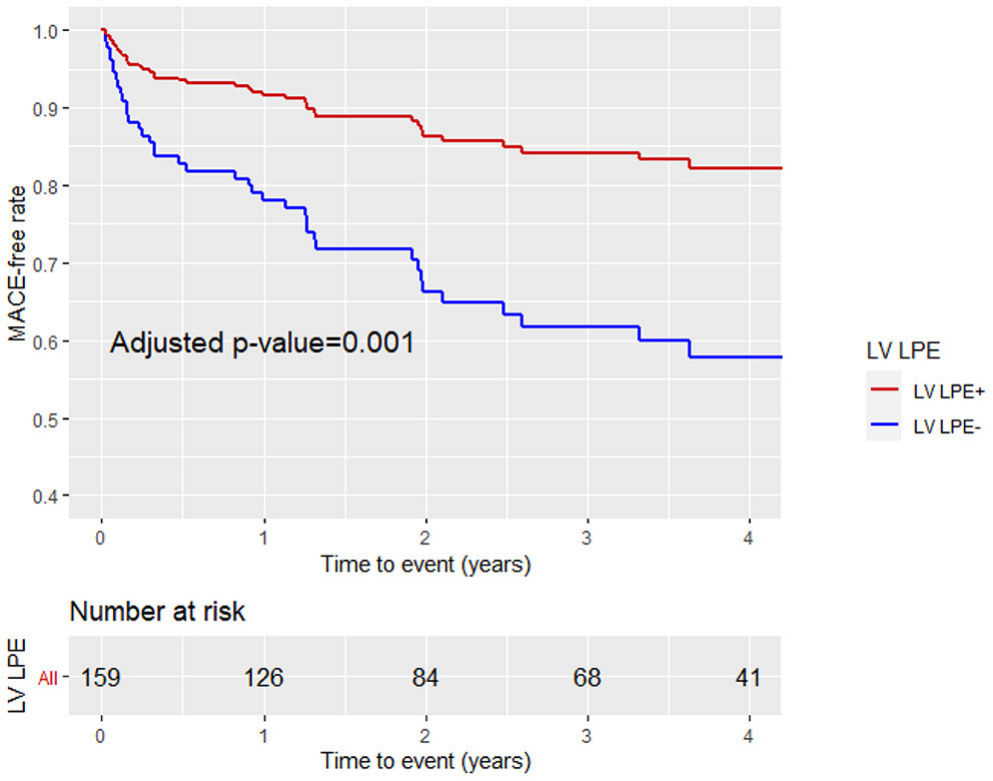
Kaplan-Meier curve analysis with the MACE-free rate on the y-axis (events) vs. time (years) on the x-axis stratified by LV pericardial involvement (LV LPE+ or LV LPE–) after adjustment for age, gender, LGE score, and MVO% of LV mass.

A multivariable Cox proportional hazard model adjusted for age, gender, LGE score, and MVO% demonstrated that LPE+, involving more than one pericardial segment, was associated with a significantly lower MACE rate [HR 0.46, 95% CI (0.25–0.86), *p* = 0.02] (Table 4; Figure 6). Pericardial involvement adjacent to the LV (LV LPE) was also associated with a significantly lower MACE rate [HR 0.39, 95% CI (0.21–0.7), *p* = 0.002] (Figure 7).

**Table 4:** A multivariable Cox proportional hazard model for predicting MACE, adjusted for age, gender, LGE score, and MVO % of LV mass.

**Variables**	**Hazard ratio**	**95% Confidence interval**	***P*-value**
Age, years	1.02	0.98–1.05	0.30
Male gender	1.12	0.33–3.80	0.85
LGE score	1.05	1.01–1.09	0.01
MVO% of LV mass	1.06	0.90–1.13	0.06
LPE+	0.46	0.25–0.86	0.01
LPE– (No LPE.)	2.17	1.60–4.01	0.01

*LGE, late Gadolinium enhancement; MVO, microvascular obstruction; LPE, late pericardial enhancement*.

The integrated approach for diagnosing post-MI pericarditis (PMIP), which combines the CMR findings of LPE together with either elevated CRP levels and/or the presence of pericardial effusion, demonstrates that 78 patients (41.7%) had PMIP. No significant differences in demographics, baseline characteristics and co-morbidities, were found between the two groups (Table 5). PMIP+ patients have significantly higher levels of inflammation and myocardial injury biomarkers (leucocyte count, CRP, CPK, and Troponin) (Table 5).

**Table 5:** Baseline characteristics of patients with or without post-MI pericarditis (CMR evidence of LPE plus either elevated CRP and/or pericardial effusion).

	***N* = 187**	**PMIP+**	**PMIP–**	***p*-value**
		**78 (41.7%)**	**109 (58.3%)**	
Age, years, mean ± sd	57.1 ± 10.5	57.5 ± 10.4	56.9 ± 10.6	0.65
Male gender, *N* (%)	171 (91.4%)	75 (96.2%)	96 (88.1%)	0.09
Active smoker, *N* (%)	79 (42.2%)	32 (41.0%)	47 (43.1%)	0.89
Hypertension, *N* (%)	58 (31%)	21 (26.9%)	37 (33.9%)	0.38
Diabetes mellitus, *N* (%)	27 (14.4%)	9 (11.5%)	18 (16.5%)	0.45
Dyslipidemia, *N* (%)	73 (39.0%)	24 (30.8%)	49 (45.0%)	0.07
Family history of IHD, *N* (%)	60 (32.1%)	26 (33.3%)	34 (31.2%)	0.88
Pain to balloon (h, median [IQR])	2.5 [2.0, 5.0]	2.5 [2.0, 5.5]	2.5 [2.0, 5.0]	0.65
WBC count*, K/μl (mean ± sd)	11.7 ± 3.9	13.0 ± 4.5	10.8 ± 3.1	<0.01
Maximal CRP**, mg/L, (median [IQR])	16.7 [5.5, 35.5]	32.7 [16.5, 58.4]	6.7 [3.8, 18.7]	<0.01
Maximal CPK (U/L, median [IQR])	1,534 [776, 3,040]	2,376 [1,262, 3,434]	1,050 [447, 2,550]	<0.01
Maximal TROPONIN (Micrg/L, median [IQR])	53.0 [17.3, 80.0]	64.5 [31.7, 80.0]	31.0 [7.9, 78.0]	<0.01
LVEF% on first echocardiography post-PPCI, mean ± sd	45.4 ± 9.6	43.8 ± 8.8	46.5 ± 9.9	0.05

A multivariable Cox proportional hazard model adjusted for age, gender, LGE score, and MVO% demonstrated that PMIP, as suggested in the integrated approach, was also associated with a significantly lower MACE rate [HR 0.46, 95% CI (0.23–0.91), *p* = 0.02] (Table 6).

**Table 6:** A multivariable Cox proportional hazard model for predicting MACE, adjusted for age, gender, LGE score, and MVO % of LV mass.

**Variables**	**Hazard ratio**	**95% Confidence interval**	***P*-value**
Age, years	1.01	0.98–1.04	0.40
Male gender	1.2	0.34–4.16	0.76
LGE score	1.05	1.01–1.09	<0.01
MVO% of LV mass	1.05	0.99–1.12	0.08
PMIP+	0.46	0.23–0.91	0.02
PMIP– (no PMIP)	2.14	1.1–4.3	0.02

## Discussion

The present CMR study demonstrated an unexpectedly high incidence of PISTEMI. Late pericardial enhancement (LPE+), the MRI manifestation of pericardial involvement, was documented in 77.5% of the patients on early post-STEMI. Cardiac MRI LPE was documented adjacent to the injured and non-injured LV myocardium, the RV, and both atria.

Post-myocardial infarction pericarditis is traditionally classified as early and late (Dressler syndrome). Its reported incidence has markedly declined in the reperfusion era from 20% to <5% (1–6). Dressler syndrome almost disappeared in the reperfusion era occurring in <0.1% of patients (12). As per current clinical practice, pericarditis is diagnosed by the presence of chest pain, pericardial friction rub, typical ECG changes, and echocardiographic evidence of pericardial effusion (2, 3, 6). Cardiac MRI is considered the modality of choice for myocardial and pericardial characterization, offering superior imaging compared with echocardiography (7–9). However, routine pericardial imaging using CMR is not performed and is recommended only in equivocal cases (13). Pericardial enhancement on the CMR LGE series is considered as evidence of active pericardial inflammation, with reported sensitivity ranging from 94 to 100% (7–9, 14, 15).

In the current study, clinical evidence of pericarditis was present in only 13/187 patients (6.9%), while PISTEMI CMR evidence was documented in 145/187 patients (77.5%).

In addition, we documented a strong correlation between PISTEMI and infarct severity. A similar correlation was reported both in the pre and post-reperfusion era. However, the reported incidence of post-STEMI pericarditis was much lower in comparison (4). Lador et al. reported post-STEMI pericarditis as relatively rare, with larger infarct size and worse short-term outcomes (5). The currently accepted concept is that post-STEMI pericarditis has become infrequent since early reperfusion (4, 5). Our CMR-based data documented early pericardial involvement in over 3/4 of our STEMI cohort. The utilization of CMR as the imaging modality of choice in our study, instead of the classical approach to post STEMI pericarditis diagnosis, explains this significant difference in prevalence. All patients in our cohort underwent early CMR, which, as stated above, is highly sensitive in detecting pericardial involvement, while the reports mentioned above are based on the combination of clinical and echocardiographic data only (2–6).

A single group of investigators reported the utilization of CMR in pericardial involvement diagnosis in two letters to the editor (16, 17). In accordance with our findings, these authors also found an increased incidence of pericardial involvement in STEMI patients, correlating with infarct size and transmurality (16). These reports defined pericardial injury as the presence of pericardial effusion and/or pericardial enhancement on CMR. The reported incidence of pericardial injury was 48%, while LPE was present in only 30% of their patients. Our study, which defined PISTEMI by the presence of pericardial enhancement, demonstrated a higher pericardial enhancement incidence of 77.5%. This discrepancy is related to the fact that the current study assessed the pericardium globally, addressing the pericardium adjacent to all four cardiac chambers. In contrast, these previous reports addressed only the pericardium adjacent to the left ventricle. In addition, differences in LGE acquisition parameters, including in-plane resolution (which were not specified in the aforementioned reports), could also contribute to this discrepancy (16, 17). The present study documented a meaningful correlation between LPE presence and infarct extent and severity. This correlation was also documented in the previous letters to the editor (16, 17). As mentioned, we have documented pericardial involvement adjacent to all four cardiac chambers, with no distinct correlation to the segmental location of the injured myocardial segments. To the best of our knowledge, this is the first report describing a global pericardial response early after STEMI. These findings might suggest that the pericardial involvement is not just an indicator of infarct severity but a more complex process related to other factors.

Post-STEMI pericarditis in the pre-reperfusion era was associated with increased long-term mortality, although it was not found to be an independent prognostic factor in follow-up studies (1–4, 6). Our study is the first CMR-based series reporting follow-up data concerning pericardial involvement in STEMI patients. As expected, patients with increased LGE and MVO sustained more MACE events. In contrast, patients with PISTEMI, defined as LPE+, had a better long-term prognosis with significantly lower MACE incidence at a median follow-up of 3 years. In addition, patients with CMR evidence of LV LPE had significantly better outcomes than those without LV LPE. While the current hypothesis-generating manuscript is not designed to provide a pathophysiological explanation to the reported findings, we assume that regenerative/reparative processes involving the left ventricle are more likely to be correlated to prognosis than the other myocardial chambers. This unexpected result raises the question of whether pericardial enhancement should still be considered a complication of acute MI.

Moreover, our data introduces a novel idea suggesting that PISTEMI results from a physiologic rather than a pathologic mechanism triggered by the myocardial injury. Experimental animal studies focusing on myocardial damage in Zebrafish and low mammalians have recently suggested that cardiac injury may activate the epicardium as a source of a regenerative or/and reparative process (18–21). A similar mechanism was not demonstrated in higher mammalians. In addition, a hint that a reparative myocardial mechanism still exists in the human heart may be found in two recent papers in pediatric patients, reporting better outcomes following cardiac surgery and viral myocarditis in children with increased circulating micro- RNA (22, 23). The presence of elevated CRP in the LPE+ group (and LV LPE+) in our study might additionally suggest that pericardial enhancement is in part a result of an immune reaction. Recently, a possible benefit from anti-inflammatory drug administration (Colchicine) in acute MI was reported (24). Close attention to the endpoint events in this study emphasizes that the anti-inflammatory treatment lowered vascular (strokes and urgent hospitalization for angina) rather than myocardial complications (24). Indeed, previous studies have warned against the utilization of anti-inflammatory drugs (Methylprednisolone and Indomethacin) in acute MI. Both drugs were associated with scar thinning, infarct expansion, and ventricular functional impairment (25–27).

Thus, our observation of frequent PISTEMI, together with those mentioned above in experimental and clinical studies, raises the challenging hypothesis that pericardial involvement is a part of a yet undefined residual myocardial repair process in the human heart. The current study did not measure specific biochemical factors and therefore cannot support this assumption with a defined physiological mechanism.

The major clinical implication of our findings is the clear understanding that pericardial involvement detected by CMR is common, occurring in more than 3/4 of STEMI patients. Thus, it should not be regarded as a post STEMI complication.

Moreover, this study documented that pericardial involvement was associated with pericardial effusion detected by echocardiography in only 3% of the cases. This is of high clinical importance since it challenges the traditional criteria of pericardial involvement diagnosis, which rely mainly on echocardiographic evidence of pericardial effusion. Cautioning that CMR detection of pericardial involvement should not be “automatically” considered as a clinical complication.

## Limitations

This is a retrospective single-center study with the related inherent limitations. The study cohort included scans performed both on 1.5 and 3 T MR scanners. Still, there was no significant difference between scanner field strength and the documented LPE presence.

Although CMR offers high spatial resolution on LGE images, there is an inability to separate the epicardial and parietal layers due to their anatomic proximity. Since the thickness of pericardium is usually <4 mm and the LPE signal is subtle, LPE quantification (similar to LGE quantification) cannot be performed. Compared to acute inflammatory pericarditis, pericardial involvement is local rather than global, rendering T2 sequences non-contributing to pericardial involvement diagnosis.

Factors involved in myocardial repair described in the animal studies were not studied in the current study.

## Conclusions

The current CMR study demonstrated that early pericardial involvement detected by CMR is common, occurring in more than 3/4 of STEMI cases, involving the pericardium adjacent to all four cardiac chambers. PISTEMI is correlated with the extent and severity of the myocardial injury. PISTEMI is associated with a decrease in the major adverse events rate on follow-up.

## Data Availability Statement

The raw data supporting the conclusions of this article will be made available by the authors, without undue reservation.

## Ethics Statement

The studies involving human participants were reviewed and approved by Sheba IRB-Helsinki Committee (International Review Board for human and animal trials) 5897-19-SMC. Written informed consent for participation was not required for this study in accordance with the national legislation and the institutional requirements.

## Author Contributions

EM and OG: conceptualization, data curation, project administration, analysis, supervision, visualization, validation, methodology, software, writing-original draft, review, and editing. YB and EK: conceptualization, supervision, CMR analysis, investigation, and validation. DO: methodology, software, writing-original draft, review, and editing. AF: methodology, software, analysis, supervision, and validation. SN, IM, AS, and RBein: conceptualization, investigation, supervision, and validation. RBeig: supervision, investigation, and validation. RG: CMR analysis, investigation, and validation. ES and SM: conceptualization, data curation, analysis, supervision, validation, writing-review, and editing. All authors contributed to the article and approved the submitted version.

## Conflict of Interest

The authors declare that the research was conducted in the absence of any commercial or financial relationships that could be construed as a potential conflict of interest.

## Publisher's Note

All claims expressed in this article are solely those of the authors and do not necessarily represent those of their affiliated organizations, or those of the publisher, the editors and the reviewers. Any product that may be evaluated in this article, or claim that may be made by its manufacturer, is not guaranteed or endorsed by the publisher.

## Abbreviations

CMR: cardiac magnetic resonance
STEMI: ST-segment elevation myocardial infarction
PISTEMI: pericardial involvement in STEMI
AHA: American Heart Association
LVEF: left ventricular ejection fraction
RVEF: right ventricular ejection fraction
LGE: late Gadolinium enhancement
MVO: microvascular obstruction
LPE: late pericardial enhancement
MACE: major adverse cardiac event.
