# Supplementary material for: Pericardial Involvement in ST-Segment Elevation Myocardial Infarction as Detected by Cardiac MRI
Source: Front Cardiovasc Med. 2022 Feb 24;9:752626. doi: 10.3389/fcvm.2022.752626 (PMC8911035; doi:10.3389/fcvm.2022.752626)
Supplement: Supplementary file 1 [file Table_1.docx]

**Appendix**

**Table 1S***:* Baseline characteristics of patients with or without left ventricle pericardial involvement only (LV LPE) on CMR.

|  | **All Patients** | **LV LPE +** | **LV LPE -** | **P-value** |
| --- | --- | --- | --- | --- |
|  | N=187 | 120 (64.1%) | 67 (35.8%) |  |
| Age, years (mean ± sd) | 57.2 ±10.5 | 57.2 ± 9.8 | 57.1 ± 11.7 | 0.98 |
| Male gender, N (%) | 171 (91.4%) | 111 (92.5%) | 60 (89.6%) | 0.67 |
| Active Smoker, N (%) | 79 (42.2%) | 52 (43.3%) | 27 (40.3%) | 0.8 |
| Hypertension, N (%) | 58 (31%) | 33 (27.5%) | 25 (37.3%) | 0.22 |
| Diabetes Mellitus, N (%) | 27 (14.4%) | 13 (10.8%) | 14 (21%) | 0.09 |
| Dyslipidemia, N (%) | 73 (39%) | 46 (38.3%) | 27 (40.3%) | 0.91 |
| Family History of IHD, N (%) | 60 (32%) | 42 (35%) | 18 (26.9%) | 0.32 |
| Pain to Balloon (hours, median [IQR]) | 2.5 [2,5] | 2.5 [2, 5] | 2.5 [2,4] | 0.59 |
| CRP Maximal (Mg/L, median [IQR]) | 16.8 [5.5, 35.5] | 23.6 [6.6, 45.7] | 7.3 [3.3, 20] | 0.002 |
| Maximal CPK (U/L, median [IQR]) | 1534  [776,3040] | 1922  [1037,3275] | 900  [352, 2357] | <0.001 |
| Maximal Troponin ( Micrg/L, median [IQR]) | 53 [17, 80] | 60 [24, 80] | 25 [7.1, 80] | 0.025 |
| LVEF % on first Echocardiography post PPCI (mean ± sd) | 45 ± 9.6 | 43.7 ± 8.9 | 48.5 ± 10 | 0.001 |
| Length of stay | 5.45 ± 2.1 | 5.4 ± 2.1 | 5.5 ± 1.9 | 0.67 |
