# Supplementary material for: Pericardial Involvement in ST-Segment Elevation Myocardial Infarction as Detected by Cardiac MRI
Source: Front Cardiovasc Med. 2022 Feb 24;9:752626. doi: 10.3389/fcvm.2022.752626 (PMC8911035; doi:10.3389/fcvm.2022.752626)
Supplement: Supplementary file 2 [file Table_2.docx]

**Table 2S**

1. Post-discharge clinical follow-up in patients with or without LPE involving more than one pericardial segment (LV, RV LA, RA)

| MACE events on follow-up | LPE + *  (23/99)** | LPE –  (23/60) |
| --- | --- | --- |
|  |  |  |
| Recurrent myocardial infarction | 6/99 | 3/60 |
| Acute stroke | 2/99 | 1/60 |
| Acute coronary syndrome necessitates urgent hospitalization and\or percutaneous coronary intervention, | 7/99 | 8/60 |
| Hospitalization for heart failure | 7/99 | 11/60 |
| Cardiovascular death | 1/99 | 0/60 |

* LPE+ involving more than one pericardial segment

** Post-discharge clinical follow-up was available 159/187 patients. Of them, 99/159 with LPE+ involved more than one pericardial segment.

**B.** Post-discharge clinical follow-up in patients with or without LPE involving only the LV.

| MACE events on follow-up | LV LPE*  (22/101) ** | LV LPE  (24/58) |
| --- | --- | --- |
|  |  |  |
| Recurrent myocardial infarction | 6/101 | 3/58 |
| Acute stroke | 1/101 | 2/58 |
| Acute coronary syndrome necessitates urgent hospitalization and\or percutaneous coronary intervention, | 6/101 | 9/58 |
| Hospitalization for heart failure | 8/101 | 10/58 |
| Cardiovascular death | 1/101 | 0/58 |

* Pericardial involvement adjacent to the LV (LV LPE)

** Post-discharge clinical follow-up was available 159/187 patients. Of them, 101/159 with LV LPE+.
